# A functional *MMP-9*-1562C>T polymorphism, MMP-9 serum levels and nephrolithiasis risk in a southern Chinese population

**DOI:** 10.3389/fmed.2023.1175798

**Published:** 2023-06-02

**Authors:** Qi Ding, Cheng Cao, Ying Shi, Zhijiang Fan, Feng Li, Wenjian Tu, Xiaohua Jin, Hailiang Zhu, Bo Fan

**Affiliations:** ^1^Department of Urology, The First People’s Hospital of Changshu, The Changshu Hospital Affiliated to Soochow University, Changshu, China; ^2^Department of Gastroenterology, The First People’s Hospital of Changshu, The Changshu Hospital Affiliated to Soochow University, Changshu, China

**Keywords:** *MMP-9*, serum levels, genetic susceptibility, nephrolithiasis, SNP

## Abstract

**Background:**

The role of matrix metalloproteinase 9 (MMP-9) in the pathophysiology of chronic kidney disease (CKD), which is associated with a nearly two-fold greater risk for urinary calculi compared to people without CKD, has been demonstrated. The aim of the research is to evaluate the association between *MMP-9*-1562C>T polymorphism, MMP-9 serum levels and nephrolithiasis risk.

**Methods:**

A hospital-based case-control study involving 302 kidney stone patients and 408 controls without kidney stone from southern China was conducted. Sanger sequencing was used to genotype the *MMP-9*-1562C>T polymorphism. The serum MMP-9 was measured in 105 kidney stone patients and 77 controls by enzyme-linked immunosorbent assay.

**Results:**

Compared to the control group, the CT genotype was more frequent in nephrolithiasis patients (adjusted OR = 1.60, 95% CI = 1.09–2.37: the risk of developing nephrolithiasis in individuals with CT genotype compared to CC genotype). Moreover, there was also a higher frequency of CT/TT genotypes among patients with nephrolithiasis (adjusted OR = 1.49, 95% CI = 1.02–2.19: the risk of developing nephrolithiasis in individuals with CT/TT genotypes compared to CC genotype). The risk remained for the subgroups of patients aged >53, smokers with pack-years of smoking >20, non-drinkers, non-diabetic patients, patients with hypertension, recurrent episodes and calcium oxalate stones (OR = 2.26, 95% CI = 1.31–3.91; OR = 5.47, 95% CI = 1.10–27.30; OR = 1.76, 95% CI = 1.14–2.72; OR = 1.54, 95% CI = 1.03–2.30; OR = 1.97, 95% CI = 1.01–3.82; OR = 1.67, 95% CI = 1.06–2.62; OR = 1.54, 95% CI = 1.02–2.32, respectively). Biochemical parameters did not differ between genotypes. Compared to controls (18.57 ± 5.80 ng/mL), nephrolithiasis patients had significantly higher serum MMP-9 levels (30.17 ± 6.78 ng/mL, *p* < 0.001). The serum MMP-9 levels of patients with CT/TT genotypes of *MMP-9*-1562C>T were significantly higher than those with CC genotype (32.00 ± 6.33 vs. 29.13 ± 6.85 ng/mL, *p* = 0.037).

**Conclusion:**

The *MMP-9*-1562C>T polymorphism in association with its soluble protein increased the risk of kidney stone, thus suggesting it could be used as a susceptibility biomarker for nephrolithiasis. Further functional studies and larger studies that include environmental exposure data are needed to confirm the findings.

## Introduction

Nephrolithiasis is a worldwide disease that seriously threatens human health with a global prevalence rate of 9% in adults (1). Moreover, there is a 50% risk that nephrolithiasis will recur within five to ten years, and 75% at twenty years after diagnosis (2, 3). Nephrolithiasis can have devastating effects on a person’s health, significantly affecting their daily life and work, and contributing to considerable economic burdens.

Numerous causes have been suggested for nephrolithiasis, including a family history of the disease, environmental exposure, hypercalciuria and dietary habits. Despite many researches, it was still not clear about the etiology of nephrolithiasis formation. A study has demonstrated that twins who were monozygotic had a higher concordance rate of kidney stones than twins who were dizygotic, suggesting genetic susceptibility factors may play a role (4).

In humans, matrix metalloproteinases (MMPs) account for a family of zinc metalloendopeptidases which have been correlated to both pathological and physiological events involving the breakdown of extracellular matrix (ECM) and the induction of fibrosis (5). An important member of MMPs, MMP-9, interacts with many biological processes, including human growth and development. Furthermore, MMP-9 plays a role in atherosclerosis pathology, such as breakdown of ECM, inflammatory cells infiltration, and plaque rupture (6). The damage to the kidney leads to inflammatory cells infiltration, including macrophages and lymphocytes. This process involves the release of inflammatory mediators from damaged and inflammatory kidney cells, including tumor necrosis factors (TNFs) and growth factors (GFs) (7, 8), which aggravates kidney inflammation and activates relevant signaling pathways, promoting renal interstitial fibrosis (9, 10). MMP-9 is critical to the process mentioned above. During renal fibrosis which is the main pathological changes of chronic kidney disease (CKD), transcription levels of *MMP-9* mRNAs increase as a result of abnormal activation and interaction of multiple cell signaling pathways (11, 12). In addition, among 21,474 patients with CKD, urinary calculi were 1.91 times more likely to occur in comparison with controls without CKD (13).

In humans, the *MMP-9* gene has 13 exons and 12 introns and is located on the long arm of chromosome 20 (20q11.1–13.1) (14). Researchers have found that the C>T functional polymorphism (rs3918242) located at-1562 bp of the *MMP-9* gene promoter may be associated with a variety of diseases including cancer, stroke and sepsis (15–17).

To date, there was one study investigating the association between the *MMP-9*-1562C>T polymorphism and nephrolithiasis risk in Malaysians. The researchers discovered that individuals with TT genotypes were more likely to develop nephrolithiasis (18). As far as we know, the polymorphism in nephrolithiasis has not been reported in Chinese populations. Considering the role of MMP-9 in nephrolithiasis, we hypothesized that *MMP-9*-1562C>T was linked to nephrolithiasis risk in Chinese subjects. To further assess the functional impact of the *MMP-9*-1562C>T polymorphism on nephrolithiasis development, we also examined the link between serum MMP-9 levels and risk of nephrolithiasis.

## Materials and methods

### Study subjects

The ethics committee of the Changshu Hospital Affiliated to Soochow University approved our study. We performed a case-control study which consisted of 302 nephrolithiasis patients and 408 controls following the approved guidelines. Briefly, we recruited patients from the Changshu Hospital Affiliated to Soochow University who had confirmed nephrolithiasis by B-ultrasound, X-ray and CT from January 2021. Patients with cancer, hyperparathyroidism, and stroke were excluded from the study. The control group consisted of healthy individuals without kidney stones who received routine physical examination at the same hospital. During recruitment, informed consent was signed by all participants and details such as age, sex, race, smoking and drinking habits were collected. As a measure of cumulative dose, pack-years of smoking (cigarettes per day divided by 20) × (years of smoking) were calculated. Drinking more than 3 times a week for at least half a year was identified as habitual drinkers, while the rest were identified as none-drinkers. Face-to-face interviews were conducted to collect individual demographic information, followed by the collection of a 5 mL venous blood sample from each participant.

### Genotyping

A Concert DNA Blood Extraction kit was used to extract genomic DNA from blood cells (Concert Biotechnology Co., Ltd., Xiamen, China). The *MMP-9*-1562C>T polymorphism was genotyped by Sanger sequencing.

### Enzyme-linked immunosorbent assay

An analysis of serum samples from 105 kidney stone patients and 77 controls was performed. We centrifuged the serum for 10 min at 2000 rpm after it clotted for half an hour at 4°C. The serum plasma samples were isolated and stored at −80°C before enzyme-linked immunosorbent assay (ELISA) testing. We used MMP-9 ELISA kits following the manufacturer’s instructions (Enzyme-linked Biotechnology Co., Ltd., Shanghai, China). The concentration of MMP-9 was obtained based on the comparison the O.D. of the samples with standard curves.

### Stone composition analysis

LILR-20 (Lanmode scientific instrument Co., Ltd., Tianjin, China) was used to assess stone composition. A skilled technician interpreted FT-IR spectra to identify specific absorption peaks to determine the qualitative composition of stone samples. When a stone was made up of multiple compounds, we calculated the ratios of different absorbencies at fixed wave numbers, and quadratic equations were used to determine relative abundances of compounds from mixtures that had known compositions (19, 20). It was considered that calculi consist of one element (calcium oxalate or calcium phosphate) when its percentage exceeded 95%. The stone was considered to be mixed if both salts were present in greater than 5% concentrations.

### Statistical analysis

The student’s *t*-test, Pearson’s *χ*^2^ test or Fisher’s exact test were applied to detect the differences in the selected variables and distributions of demographic characteristics between cases and controls. Using adjusted odds ratios (ORs) and 95% confidence intervals (CIs) from unconditional logistic regression, we estimated the association between the nephrolithiasis risk and genotypes. All ORs were adjusted for age, sex, pack-years of smoking, drinking, hypertension and diabetes status. Among controls, genotype frequencies were calculated using Hardy–Weinberg equilibrium (HWE). All statistical analyses were performed with Statistics Analysis System software (Version 9.4; SAS Institute, Inc., Cary, NC, United States) and the differences were considered statistically significant when two-sided *p* was <0.05.

## Results

### Characteristics of study subjects

As shown in the Table 1, there was a good match between the age of the cases and the controls. The average age was 53.01 ± 13.29 years for the cases and 53.01 ± 13.17 years for the controls (*p* = 0.997). Among the two groups, there was a similar sex composition (*p* = 0.181). The BMI values, proportions of hypertension, diabetes, smoking, and alcohol drinking were also similar between these two groups. However, the cases had a larger proportion of high BMI (BMI >24 kg/m^2^) individuals than the controls (56.62% vs. 48.53%, *p* = 0.033). Furthermore, 18.54% of cases had a positive family history of urolithiasis, significantly higher than controls (9.37%) (*p* < 0.001).

**Table 1:** The characteristics description of kidney stone patients and controls.

Variables	Cases (*n* = 302) (%)	Controls (*n* = 408) (%)	*p*-value^a^
Age (years)	53.01 ± 13.29	53.01 ± 13.17	0.997
≤53	152 (50.33)	181 (44.36)	0.115
>53	150 (49.67)	227 (55.64)	
Sex			
Male	203 (67.22)	251 (61.52)	0.118
Female	99 (32.78)	157 (38.48)	
BMI (kg/m^2^)	24.58 ± 2.99	24.23 ± 3.40	0.147
≤24	131 (43.38)	210 (51.47)	**0.033**
>24	171 (56.62)	198 (48.53)	
Smoking status			
Never	196 (64.90)	288 (70.59)	0.108
Ever	106 (35.10)	120 (29.41)	
Pack-years of smoking			
0	196 (64.90)	290 (71.08)	0.0541
0–20	54 (17.88)	73 (17.89)	
>20	52 (17.22)	45 (11.03)	
Drinking status			
Never	236 (78.15)	327 (80.15)	0.515
Ever	66 (21.85)	81 (19.85)	
Family history of urolithiasis			
Yes	56 (18.54)	26 (9.37)	**<0.001**
No	246 (81.46)	382 (93.63)	
Hypertension			
Yes	111 (36.75)	137 (33.58)	0.380
No	191 (63.25)	271 (66.42)	
Diabetes			
Yes	31 (10.26)	41 (10.05)	0.925
No	271 (89.74)	367 (89.95)	

Bold values indicate significant difference.

a *t*-test for age distributions between the cases and controls; two-sided chi-squared test for others selected variables between the cases and controls.

### Genotype distribution of the *MMP-9*-1562C>T polymorphism between the cases and controls

Table 2 shows the genotype frequencies of the *MMP-9*-1562C>T polymorphism among nephrolithiasis patients and controls, along with its association with nephrolithiasis risk. In the control group, genotype frequencies were consistent with HWE (*p* = 0.082). The frequencies of the CC, CT, and TT genotypes were 77.81%, 21.52%, 0.67%, respectively, among the cases; and they were 83.82%, 14.71%, 1.47%, respectively, among the controls (*p* = 0.048). Compared to the control group, the CT genotype was more frequent in nephrolithiasis patients (adjusted OR = 1.60, 95% CI = 1.09–2.37: the risk of developing nephrolithiasis in individuals with CT genotype compared to CC genotype). Moreover, there was also a higher frequency of CT/TT genotypes among patients with nephrolithiasis (adjusted OR = 1.49, 95% CI = 1.02–2.19: the risk of developing nephrolithiasis in individuals with CT/TT genotypes compared to CC genotype). However, there was no difference in allele distribution between the two groups (*p* = 0.105).

**Table 2:** Genotype frequencies of *MMP-9*-C1562T among cases and controls and their associations with kidney stone risk.

Genotypes	Cases (*n* = 302)		Controls (*n* = 408)^a^		*p*-value^b^	OR (95% CI)	*p*-value^c^	Adjusted OR (95% CI)^c^
	*n*	%	*n*	%				
CC	235	77.81	342	83.82	**0.048** ^d^	1.00 (reference)	**0.048** ^d^	1.00 (reference)
CT	65	21.52	60	14.71		**1.57 (1.07–2.32)**		**1.60 (1.09–2.37)**
TT	2	0.67	6	1.47		0.49 (0.10–2.43)		0.46 (0.09–2.34)
CT/TT	67	22.19	111	16.18	**0.043**	**1.47 (1.01–2.16)**	**0.039**	**1.49 (1.02–2.19)**
*p*_trend_					**0.043**			
Allele								
C	535	88.58	744	91.18		1.00 (reference)		
T	69	11.42	72	8.82	0.105	1.33 (0.94–1.89)		

Bold values indicate significant difference.

a The observed genotype frequencies among the control subjects were in agreement with the Hardy–Weinberg equilibrium (*χ*^2^ = 3.019, *p* = 0.082).

b Two-sided chi-squared test for either genotype distributions or allele frequencies between the cases and controls.

c Adjusted for age, sex, pack-years of smoking, drinking status, hypertension and diabetes in logistic regression model.

d Fisher’s exact test.

Furthermore, stratified analyses of age, sex, BMI, pack year of smoking, drinking habits, hypertension status, diabetes status and family history were conducted. As presented in Table 3, based on CC genotype as reference, there were also increases in nephrolithiasis risk for CT/TT genotypes in subgroups of patients aged >53 (adjusted OR = 2.26, 95% CI = 1.31–3.91, *p* = 0.003), smokers with pack-years of smoking >20 (adjusted OR = 5.47, 95% CI = 1.10–27.30, *p* = 0.039), non-drinkers (adjusted OR = 1.76, 95% CI = 1.14–2.72, *p* = 0.011), patients with hypertension (adjusted OR = 1.97, 95% CI = 1.01–3.82, *p* = 0.045) and non-diabetic patients (adjusted OR = 1.54, 95% CI = 1.03–2.30, *p* = 0.035).

**Table 3:** Stratification analyses between *MMP-9-*C1562T genotypes and risk of kidney stone in cases and controls.

Characteristics	Cases (*n* = 302)		Controls (*n* = 408)		*p*-value^a^	OR (95% CI)^a^
	CC	CT/TT	CC	CT/TT		
Age (years)						
≤53	122	30	146	35	0.952	1.02 (0.58–1.78)
>53	113	37	196	61	**0.003**	**2.26 (1.31–3.91)**
Sex						
Male	156	47	208	43	0.112	1.47 (0.91–2.36)
Female	79	20	134	23	0.283	1.46 (0.73–2.90)
BMI						
≤24	95	36	170	40	0.061	1.66 (0.98–2.82)
>24	140	31	172	26	0.181	1.48 (0.83–2.64)
Smoking status						
Never	155	41	240	49	0.295	1.28 (0.81–2.04)
Ever	80	26	102	17	0.077	1.87 (0.93–3.76)
Packyear of smoking						
0	155	41	240	49	0.295	1.28 (0.81–2.04)
0–20	39	15	59	15	0.211	1.76 (0.73–4.27)
>20	41	11	43	2	**0.039**	**5.47 (1.10–27.30)**
Drinking status						
Never	182	54	279	48	**0.011**	**1.76 (1.14–2.72)**
Ever	53	13	63	18	0.448	0.72 (0.31–1.69)
Hypertension						
Yes	84	27	118	19	**0.045**	**1.97 (1.01–3.82)**
No	151	40	224	47	0.318	1.28 (0.79–2.06)
Diabetes						
Yes	27	4	35	6	0.488^b^	0.58 (0.12–2.71)
No	208	63	307	60	**0.035**	**1.54 (1.03–2.30)**
Family history of urolithiasis						
Yes	46	10	26	0	0.959^b^	–
No	189	57	316	66	0.060	1.47 (0.98–2.20)

Bold values indicate significant difference.

a Adjusted for age, sex, pack-years of smoking, drinking status, hypertension and diabetes in logistic regression model.

b Continuous correction chi-squared test.

### Association between the *MMP-9*-1562C>T genotypes and biochemical data in patients with nephrolithiasis

To better understand the association between the polymorphism and clinical risk factors, biochemical parameters including urine pH, serum calcium, uric acid, phosphorus and creatinine were analyzed. As shown in Table 4, biochemical parameters did not differ between genotypes. Moreover, no significant differences were found in any comparison of the stratified analysis based on their levels (Table 5).

**Table 4:** Association between the *MMP-9*-C1562T genotypes and biochemical parameters in kidney stone patients.

Genotype	Urine pH	Serum calcium (mg/dL)	Serum creatinine (mg/dL)	Serum uric acid (mg/dL)	Serum phosphorus (mg/dL)
CC	6.00 ± 0.60	9.34 ± 0.88	1.05 ± 0.49	6.24 ± 1.66	3.17 ± 0.49
CT	5.92 ± 0.64	9.25 ± 0.50	1.10 ± 0.53	6.16 ± 1.69	3.21 ± 0.61
TT	6.00 ± 0.71	9.38 ± 0.57	0.89 ± 0.03	5.08 ± 1.09	2.76 ± 0.53
CT/TT	5.93 ± 0.64	9.25 ± 0.50	1.09 ± 0.52	6.12 ± 1.68	3.19 ± 0.61
*p*-value^a^	0.354	0.251	0.459	0.712	0.659
*p*-value^b^	0.996	0.954	0.656	0.323	0.233
*p*-value^c^	0.362	0.266	0.508	0.607	0.777

a *t*-test for biochemical parameters between CT and CC.

b *t*-test for biochemical parameters between TT and CC.

c *t*-test for biochemical parameters between CT/TT and CC.

**Table 5:** Association between the *MMP-*9-C1562T genotypes and abnormal biochemical indexes in kidney stone patients.

Variables	Genotype		*p*-value^a^	OR (95% CI)^a^
	CC (*n* = 235)	CT/TT (*n* = 67)		
Serum calcium				
>10.2 (mg/dL)	6 (2.55%)	2 (2.99%)	1.000^b^	1.47 (0.28–7.90)
≤10.2 (mg/dL)	229 (97.45%)	65 (97.01%)		
Serum creatinine				
>1.3 (mg/dL)	41 (17.45%)	10 (14.93%)	0.627	0.86 (0.40–1.84)
≤1.3 (mg/dL)	194 (82.55%)	57 (85.07%)		
Serum uric acid				
>7.2 (mg/dL)	68 (28.94%)	16 (23.88%)	0.415	0.74 (0.39–1.41)
≤7.2 (mg/dL)	167 (71.06%)	51 (76.12%)		
Serum phosphorus				
>4.5 (mg/dL)	1 (0.43%)	1 (1.49%)	0.395^c^	4.10 (0.24–69.04)
≤4.5 (mg/dL)	234 (99.57%)	66 (98.51%)		

a Adjusted for age, sex, pack-years of smoking, drinking status, hypertension and diabetes in logistic regression model.

b Continuous correction chi-squared test.

c Fisher’s exact test.

### Association between the *MMP-9*-1562C>T genotypes and the risk of multiple stones or recurrence in kidney stone patients

Out of the 302 patients with nephrolithiasis, 170 had a single stone, while 132 patients had multiple stones. In addition, there were 137 patients with single episode and 165 with recurrent episodes. No significant association of the SNP (single nucleotide polymorphism) with the risk of multiple stones was found. Compared to the control group, the CT/TT genotypes were more frequent in patients with recurrent episodes of nephrolithiasis (adjusted OR = 1.67, 95% CI = 1.06–2.62, *p* = 0.027: the risk of developing nephrolithiasis in individuals with CT/TT genotype compared to CC genotype) (Table 6).

**Table 6:** Association between the *MMP-9*-C1562T genotypes and multiple stones and recurrence in kidney stone patients.

Variables	CC		CT/TT		*p*-value^a^	OR (95% CI)^b^
	*n*	%	*n*	%		
Controls (*n* = 408)	342	83.82	66	16.18		1.00 (reference)
Cases (*n* = 302)						
Recurrent						
Yes (*n* = 165)	126	76.36	39	23.64	**0.027**	**1.67 (1.06–2.62)**
No (*n* = 137)	109	79.56	28	20.44	0.246	1.34 (0.82–2.22)
Multiple						
Yes (*n* = 132)	103	78.03	29	21.97	0.097	1.52 (0.93–2.49)
No (*n* = 170)	132	77.65	38	22.35	0.076	1.51 (0.96–2.37)

Bold values indicate significant difference.

a Two-sided *χ*^2^-test for abnormal biochemical indexes between CC and CT/TT.

b Adjusted for age, sex, pack-years of smoking, drinking status, hypertension and diabetes in logistic regression model.

### Association between the *MMP-9*-1562C>T genotypes and chemical stone composition

A further analysis of the relationship between *MMP-9*-1562C>T genotypes and chemical stone composition was performed. Compared with the control group, the frequency of CT/TT genotypes was significantly higher in patients with calcium oxalate stones (adjusted OR = 1.54, 95% CI =1.02–2.32: the risk of developing nephrolithiasis in individuals with CT/TT genotype compared to CC genotype) (Table 7).

**Table 7:** Association between the *MMP-9*-C1562T genotypes and chemical stone composition.

Variables	CC		CT/TT		*p*-value^a^	OR (95% CI)^a^
	*n*	%	*n*	%		
Controls (*n* = 408)	342	83.82	66	16.18		1.00 (reference)
Cases (*n* = 302)						
Chemical stone composition						
Calcium oxalate stones (*n* = 229)	177	77.29	52	22.71	**0.040**	**1.54 (1.02–2.32)**
Carbonate apatite (*n* = 30)	29	96.67	1	3.33	0.105^b^	0.18 (0.02–1.32)
Uric acid stones (*n* = 21)	16	76.19	5	23.81	0.537^b^	1.80 (0.62–5.25)
Struvite (*n* = 13)	8	61.54	5	38.46	0.083^b^	3.23 (0.99–10.46)
Calcium phosphate stones (*n* = 8)	5	62.50	3	37.50	0.260^b^	3.00 (0.66–13.70)
Xanthine (*n* = 1)	0	0	1	100	0.131^c^	–

Bold values indicate significant difference.

a Adjusted for age, sex, pack-years of smoking, drinking status, hypertension and diabetes in logistic regression model.

b Continuous correction chi-squared test.

c Fisher’s exact test.

### Association between MMP-9 serum levels and nephrolithiasis risk

**Figure 1:**
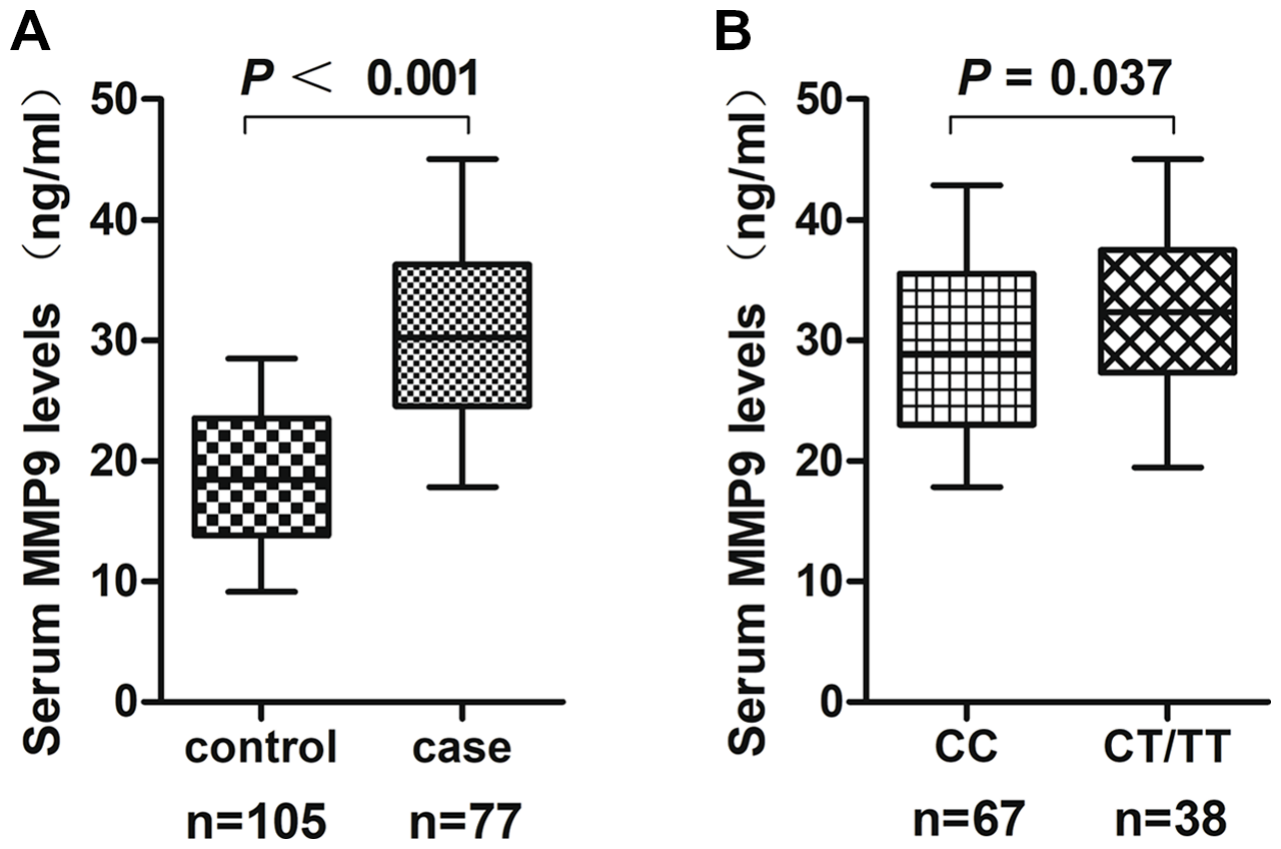
**(A)** The serum MMP-9 levels in the kidney stone patients and the controls. **(B)** The serum MMP-9 levels in the kidney stone patients stratified according to the C1562T genotype.

A total of 182 serum samples including 105 kidney stone patients and 77 controls were included in this study. The mean serum MMP-9 level was 30.17 ± 6.78 ng/mL in cases，which was significantly higher than that in controls (18.57 ± 5.80 ng/mL) (*p* < 0.001) (Figure 1A and Table 8). When the third quartiles MMP-9 level in controls (23.33 ng/mL) was used as the cutoff value, 80% (84 of 105) of the cases exceeded the value. After adjusting for age, sex, smoking pack-years, alcohol consumption, hypertension and diabetes, serum levels of MMP-9 above this level were significantly associated with nephrolithiasis risk (adjusted OR = 13.43, 95% CI = 6.19–29.14) (Table 8). In addition, serum MMP-9 levels of subjects with the CT/TT genotypes (32.00 ± 6.33 ng/mL) were significantly higher than those with the CC genotype (29.13 ± 6.85 ng/mL) (*p* = 0.037) (Figure 1B).

**Table 8:** Logistic regression analyses on associations between serum MMP-9 levels and risk of kidney stone.

Variable	Cases (*n* = 105) (%)	Controls (*n* = 77) (%)	*p*-value	OR (95% CI)^b^
Serum MMP-9 (ng/mL)	30.17 ± 6.78	18.57 ± 5.80	**<0.001** ^a^	–
≤23.33	21 (20.00)	58 (75.32)		1.00 (reference)
>23.33	84 (80.00)	19 (24.68)	**<0.001** ^b^	**13.43 (6.19–29.14)**

Bold values indicate significant difference.

a *t*-test for serum MMP-9 levels between the cases and controls.

b Adjusted for age, sex, pack-years of smoking, drinking status, hypertension and diabetes in logistic regression model.

## Discussion

We investigated the relationship between the *MMP-9*-1562C>T SNP (rs3918242) and the risk of nephrolithiasis in a southern Chinese population in this hospital-based case-control study. Comparing CT and CC genotypes frequencies between nephrolithiasis patients and controls, we found that CT genotype was significantly more prevalent in the nephrolithiasis patients; also it was revealed that the variant T allele (CT/TT) was significantly more frequent in the lithiasis population. Further stratification revealed that the risk remained for the subgroup of patients aged >53, smokers with pack-years of smoking >20, non-drinkers, non-diabetic patients, patients with hypertension, recurrent episodes and calcium oxalate stones. Compared to controls, nephrolithiasis patients had significantly higher serum MMP-9 levels. Moreover, patients with nephrolithiasis who had the CT/TT genotype had significantly higher serum MMP-9 levels than those who had the CC genotype. To the best of our knowledge, no studies have yet examined the association between the *MMP-9*-1562C>T polymorphism and nephrolithiasis in Chinese individuals.

MMPs are zinc-dependent endopeptidases that can degrade ECM components, including collagen, which was essential for tissue repair and reconstruction during inflammation (21, 22). The MMP-9 enzyme is primarily produced by neutrophils and macrophages and has been demonstrated to regulate inflammation. In addition, growth factors (GFs), cytokines and adhesion molecules are also affected by MMP-9, all of which play crucial roles in inflammation process (23–26). A link between MMP-9 and extracellular signal-regulated kinase (ERK) can be found in Schwann cells, and ERK involves in renal interstitial fibrosis, which is thought to be the final result of CKD (27). By interacting with cytokines such as tumor necrosis factors (TNFs) and GFs etc., MMP-9 contributes to the development and progression of CKD (28). Furthermore, CKD patients were found to have nearly two-fold risk of urinary calculi than individuals without CKD (13). Thus, it’s plausible that MMP-9 play a significant role in the genesis of kidney stone.

A functional C-to-T SNP (rs3918242) has been identified in the *MMP-9* gene at position 1,562, which is close to the transcription start site. The polymorphism increases mRNA, protein expression, and *MMP-9* activity in T allele carriers, whereas the C allele causes a decrease in these activities (29, 30). Consistently, in this study, a significant elevation in serum MMP-9 levels was observed in nephrolithiasis patients with the CT/TT genotypes compared with the CC genotype. Taken together, the evidence suggests that individuals with the variant T allele may gain MMP-9 function and be more prone to develop CKD, and ultimately increase the susceptibility to nephrolithiasis.

According to our findings, heavy smokers with more than 20 pack years of smoking seem to be more susceptible to developing nephrolithiasis with the variant T allele. Cigarette smoke is a complex mixture of chemical compounds, most of which has been proven to be cytotoxic, mutagenic, and oncogenic. Research has shown that cigarette smoke extract (CSE) can up-regulate the production of MMP-9 in rat vascular smooth muscle cell (31). In addition, alveolar macrophages were found to respond to CSE by over-expressing MMP-9 (32). Smokers with pulmonary tuberculosis had higher levels of MMP-9 in their bronchoalveolar lavage (33). Consistent with these findings, our study showed that heavy smokers carrying the T allele were at an increased risk of nephrolithiasis, indicating that the *MMP-9*-1562C>T polymorphism has synergistic effect with CSE in nephrolithiasis by up-regulating the expression of MMP-9. Further studies on the mechanism of MMP-9 and CSE in the formation of nephrolithiasis are warranted in the future.

It is interesting to see that the *MMP-9*-1562C/T polymorphism increased the risk of nephrolithiasis in patients with hypertension. Basic studies on hypertension have revealed that MMP-9 expression was significantly higher in conduct vessels with thicker intimas and media and the process could be blocked by MMP inhibitor (doxycycline) treatment (34, 35). Tan et al. (36) showed that patients with essential hypertension had increased MMP-9 levels that were linearly related to arterial wall stiffness. Similarly, Yasmin et al. (37) investigated the stiffness of the aorta media in individuals who had isolated systolic hypertension and concluded that MMP-9 was predictive of aortic wall stiffness. Furthermore, Cappuccio et al. (38) noticed that individuals with hypertension were more likely to develop nephrolithiasis. These findings suggest that MMP-9 could play a vital role within the pathological process of hypertension which may ultimately increase the risk of developing nephrolithiasis.

A cross-sectional study of 3 large cohort including 200,000 subjects showed that an increased risk of kidney stones was found in patients with type 2 diabetes (39). Chung et al. (40) conducted a prospective study of 94,276 individuals followed five years. They found that patients with urinary calculi had a 1.32-fold increased risk of receiving a first diagnosis of diabetes. Tests in animal models of diabetes have shown that the activity and expression of MMP-9 was significantly increased in vascular tissue and plasma (41). Conversely, we found that the CT/TT genotype was associated with an increased risk for nephrolithiasis in non-diabetic but not diabetic subjects. The possible reasons are as follows. First, the smoking rate of non-diabetic patients in the stone group was 36.16%, which was significantly higher than that in the control group (28.07%) (*p* = 0.030). However, there was no significant difference in the smoking rate of diabetic patients between the two groups (*p* = 0.239). Multiple studies have shown that CSE or smoking can increase MMP9 expression *in vitro* and *in vivo* (31–33). Thus, the higher smoking rate among non-diabetics in this study may contribute to the risk of developing kidney stone. Second, there were only 31 diabetic patients in the stone group and 41 in the control group. The statistical power is poor and may not yield true results. A larger sample of diabetic patients will be included in the follow-up to obtain more credible results on the relationship between the polymorphism and the kidney stone risk in diabetic patients.

Our study showed that the *MMP-9*-1562C>T polymorphism correlates with recurrent nephrolithiasis. The lifetime recurrence risk of nephrolithiasis among Asian countries is estimated to be 60%–80% (42). It is notable that the socioeconomic burden of recurrent stone disease is high, with direct and indirect costs of treatment exceeding five billion dollars (43). Until now, several SNPs in urokinase and vitamin D receptor (VDR) have been revealed to be related to recurrent kidney stones (44–46). Integrating these findings with those of our own will help identify those who are at risk of developing kidney stones again, lowering the health and socioeconomic costs associated with nephrolithiasis.

Compared with the control group, the frequency of CT/TT genotypes was significantly higher in patients with calcium oxalate stones. Similarly, one SNP (rs1056628) in the 3′ untranslated region of *MMP-9* was found to be associated with susceptibility to calcium oxalate stones (47). Cell experiments showed that the expression of MMP-9 increased in normal rat kidney epithelial-like (NRK-52E) cells after treatment of high concentration of calcium. The overexpression of MMP-9 could increase the expression of osteoblastic-related proteins and promote calcium crystal deposition. Knockout of MMP-9 resulted in opposite result. Calcium crystal deposition was drastically decreased in hypercalciuric rats after treatment with MMP-9 inhibitor I (48). Taken together, MMP-9 might promote the formation of calcium oxalate kidney stones through the above mechanisms. However, more researches should be done on the underlying processes of this polymorphism in calcium oxalate stones.

To date, there has only been one published report of the association between *MMP-9*-1562C>T polymorphism and nephrolithiasis risk in Malaysians. Mehde et al. (18) showed that the homozygote TT was more frequent in the nephrolithiasis patients group. Nephrolithiasis patients with CT and TT genotypes had significantly higher serum MMP-9 levels than patients with CC genotypes. Their findings are partly consistent with ours. However, we observed no correlation between the TT genotype and the risk of nephrolithiasis (OR = 0.46, 95% CI = 0.09–2.34, *p* = 0.483). Between the two study populations, these discrepancies can be attributed to differences in environmental exposure, detection methods, and ethnic background. For example, the frequencies of the CC, CT, and TT genotypes in our control were 83.82%, 14.71%, 1.47%, respectively, which were different from theirs (77.50%, 19.17%, 3.33%). On the other hand, the research by Mehde et al. contains 120 cases and 120 controls. The sample size was relatively small, with insufficient statistical test efficacy to explore the real association. Furthermore, we performed stratified analyses based on age, sex, BMI, pack year of smoking, drinking habits, hypertension status, diabetes status, family history, multiple stones, recurrence, and chemical stone composition to get more precise results.

In this study, some limitations need to be stated. First, lack of data on environmental factors made it difficult to further analyze potential correlations. It is possible that gene-environment interactions affect kidney stone risk. Second, our research studied only one SNP. It should be investigated to see if any further functional polymorphisms in the *MMP-9* gene, which have not yet been found, increased the risk of nephrolithiasis. Third, the role of MMP-9 in nephrolithiasis is not completely understood and further experimental studies are nevertheless necessary to explore the specific mechanisms for *MMP-9* polymorphism and nephrolithiasis. Fourth, given that our study was hospital-based, selection bias was inevitable and our control population might not be accurately representative of the general population. However, in this study, incident patients were recruited. Moreover, in our Chinese control subjects, genotype distribution was similar to that reported for other diseases in the literature. For example, the frequencies of CC, CT, and TT genotypes in our controls were 83.82%, 14.71%, 1.47%, respectively, which resembles those discovered by Li et al. (16) (82.8%, 16.0%, and 1.2% for the CC, CT, and TT, respectively). As well, the genotype frequencies of controls are in accordance with HWE, indicating that there is no discernible selection bias in regard to genotype distribution. Last, the proportion of individuals with CKD was not clarified in the study. Thus, we were unable to examine the relationship between CKD, kidney stones and *MMP-9*-1562C>T polymorphism in more detail. Further studies are needed to explore the interrelations among them.

In conclusion, the results of the current study demonstrated a significant association between the variation T allele of the *MMP-9*-1562C>T polymorphism with the likelihood of developing nephrolithiasis. The risk remained for the subgroup of patients aged >53, smokers with pack-years of smoking >20, non-drinkers, non-diabetic patients, patients with hypertension, recurrent episodes and calcium oxalate stones. Also, we noted that nephrolithiasis patients had serum MMP-9 levels that were considerably greater than those of controls. Patients with nephrolithiasis who carried the CT/TT genotype had serum MMP-9 levels that were notably greater compared to patients with the CC genotype. The *MMP-9*-1562C>T polymorphism may be useful as a susceptibility marker for nephrolithiasis as it’s linked to its soluble protein and enhances the possibility of developing nephrolithiasis. However, this association needs to be confirmed by further experimental studies and larger studies that include detailed information about environmental exposure.

## Data availability statement

The original contributions presented in the study are included in the article/supplementary material, further inquiries can be directed to the corresponding author.

## Ethics statement

The studies involving human participants were reviewed and approved by The ethics committee of Changshu Hospital affiliated with Soochow University. The patients/participants provided their written informed consent to participate in this study.

## Author contributions

QD, CC, YS, ZF, FL, WT, XJ, HZ, and BF contributed significantly to this work and reviewed the manuscript. QD, CC, YS, ZF, FL, WT, XJ, and HZ performed the research study and collected the samples and data. QD and YS analyzed the data. QD and BF designed the research study. QD was a major contributor in writing the manuscript. ZF and FL prepared all the tables. All authors contributed to the article and approved the submitted version.

## Funding

This study was supported by the Youth Science Funding Project of Suzhou (KJXW2020063), the Changshu Medical Science and Technology Youth Program (cswsq202102), Clinical Key Subject Construction Project Foundation of Changshu (CSZDXK202001) and Introduction Project of Clinical Medicine Expert Team for Changshu (CSYJTD202001).

## Conflict of interest

The authors declare that the research was conducted in the absence of any commercial or financial relationships that could be construed as a potential conflict of interest.

## Publisher’s note

All claims expressed in this article are solely those of the authors and do not necessarily represent those of their affiliated organizations, or those of the publisher, the editors and the reviewers. Any product that may be evaluated in this article, or claim that may be made by its manufacturer, is not guaranteed or endorsed by the publisher.

## References

[1] Khan SR Pearle MS Robertson WG Gambaro G Canales BK Doizi S. Kidney stones. Nat Rev Dis Primers. (2016) 2:16008. doi: 10.1038/nrdp.2016.8, PMID: 27188687 PMC5685519

[2] Sutherland JW Parks JH Coe FL. Recurrence after a single renal stone in a community practice. Miner Electrolyte Metab. (1985) 11:267–9. PMID: 4033604

[3] Trinchieri A Ostini F Nespoli R Rovera F Montanari E Zanetti G. A prospective study of recurrence rate and risk factors for recurrence after a first renal stone. J Urol. (1999) 162:27–30. doi: 10.1097/00005392-199907000-00007, PMID: 10379732

[4] Goldfarb DS Fischer ME Keich Y Goldberg J. A twin study of genetic and dietary influences on nephrolithiasis: a report from the Vietnam era twin (VET) registry. Kidney Int. (2005) 67:1053–61. doi: 10.1111/j.1523-1755.2005.00170.x, PMID: 15698445

[5] Galis ZS Khatri JJ. Matrix metalloproteinases in vascular remodeling and atherogenesis: the good, the bad, and the ugly. Circ Res. (2002) 90:251–62. doi: 10.1161/res.90.3.251 11861412

[6] Tamarina NA McMillan WD Shively VP Pearce WH. Expression of matrix metalloproteinases and their inhibitors in aneurysms and normal aorta. Surgery. (1997) 122:264–72. doi: 10.1016/s0039-6060(97)90017-9 9288131

[7] Boor P Floege J. Chronic kidney disease growth factors in renal fibrosis. Clin Exp Pharmacol Physiol. (2011) 38:441–50. doi: 10.1111/j.1440-1681.2011.05487.x 21276040

[8] Vielhauer V Mayadas TN. Functions of TNF and its receptors in renal disease: distinct roles in inflammatory tissue injury and immune regulation. Semin Nephrol. (2007) 27:286–308. doi: 10.1016/j.semnephrol.2007.02.004, PMID: 17533007

[9] Li L Tan J Zhang Y Han N Di X Xiao T. DLK1 promotes lung cancer cell invasion through upregulation of MMP9 expression depending on notch signaling. PLoS One. (2014) 9:e91509. doi: 10.1371/journal.pone.0091509, PMID: 24621612 PMC3951400

[10] Wiercinska E Naber HP Pardali E van der Pluijm G van Dam H ten Dijke P. The TGF-β/Smad pathway induces breast cancer cell invasion through the up-regulation of matrix metalloproteinase 2 and 9 in a spheroid invasion model system. Breast Cancer Res Treat. (2011) 128:657–66. doi: 10.1007/s10549-010-1147-x 20821046

[11] Pei G Zeng R Han M Liao P Zhou X Li Y. Renal interstitial infiltration and tertiary lymphoid organ neogenesis in IgA nephropathy. Clin J Am Soc Nephrol. (2014) 9:255–64. doi: 10.2215/cjn.01150113, PMID: 24262509 PMC3913227

[12] Tang X Zhang J Cai DH Zeng L. Effect of high glucose exposure on connective tissue growth factor expression in cultured human renal tubular epithelial cells and the role of p38MAPK pathway. J Southern Med Univ. (2009) 29:50–3. 19218111

[13] Keller JJ Chen YK Lin HC. Association between chronic kidney disease and urinary calculus by stone location: a population-based study. BJU Int. (2012) 110 (11 Pt C), E1074-1078:E1074–8. doi: 10.1111/j.1464-410X.2012.11380.x, PMID: 22934937

[14] Zhang B Henney A Eriksson P Hamsten A Watkins H Ye S. Genetic variation at the matrix metalloproteinase-9 locus on chromosome 20q12.2-13.1. Hum Genet. (1999a) 105:418–23. doi: 10.1007/s004390051124, PMID: 10598806

[15] Decock J Paridaens R Ye S. Genetic polymorphisms of matrix metalloproteinases in lung, breast and colorectal cancer. Clin Genet. (2008) 73:197–211. doi: 10.1111/j.1399-0004.2007.00946.x, PMID: 18177467

[16] Li Y Chen L Yao S Chen J Hu W Wang M. Association of polymorphisms of the matrix metalloproteinase 9 gene with ischaemic stroke in a southern Chinese population. Cell Physiol Biochem. (2018a) 49:2188–99. doi: 10.1159/000493823, PMID: 30257242

[17] Zheng C Wang J Xie S. Matrix metalloproteinase-9-1562 C/T polymorphism is associated with the risk of sepsis in a Chinese population: a retrospective study. Innate Immun. (2021) 27:260–5. doi: 10.1177/1753425921992414, PMID: 33593148 PMC8054153

[18] Mehde AA Mehdi WA Yusof F Raus RA Zainal Abidin ZA Ghazali H. Association of MMP-9 gene polymorphisms with nephrolithiasis patients. J Clin Lab Anal. (2018) 32:e22173. doi: 10.1002/jcla.22173, PMID: 28205286 PMC6817110

[19] Cohen-Solal F Dabrowsky B Boulou JC Lacour B Daudon M. Automated Fourier transform infrared analysis of urinary stones: technical aspects and example of procedures applied to carbapatite/weddellite mixtures. Appl Spectrosc. (2004) 58:671–8. doi: 10.1366/000370204872962, PMID: 15198818

[20] Maurice-Estepa L Levillain P Lacour B Daudon M. Advantage of zero-crossing-point first-derivative spectrophotometry for the quantification of calcium oxalate crystalline phases by infrared spectrophotometry. Clin Chim Acta. (2000) 298:1–11. doi: 10.1016/s0009-8981(00)00224-2, PMID: 10876000

[21] Hannas AR Pereira JC Granjeiro JM Tjäderhane L. The role of matrix metalloproteinases in the oral environment. Acta Odontol Scand. (2007) 65:1–13. doi: 10.1080/00016350600963640 17354089

[22] Sorsa T Tjäderhane L Konttinen YT Lauhio A Salo T Lee HM. Matrix metalloproteinases: contribution to pathogenesis, diagnosis and treatment of periodontal inflammation. Ann Med. (2006) 38:306–21. doi: 10.1080/07853890600800103, PMID: 16938801

[23] Bai X Bai G Tang L Liu L Li Y Jiang W. Changes in MMP-2, MMP-9, inflammation, blood coagulation and intestinal mucosal permeability in patients with active ulcerative colitis. Exp Ther Med. (2020) 20:269–74. doi: 10.3892/etm.2020.8710, PMID: 32536995 PMC7282134

[24] Bruschi F D’Amato C Piaggi S Bianchi C Castagna B Paolicchi A. Matrix metalloproteinase (MMP)-9: a realiable marker for inflammation in early human trichinellosis. Vet Parasitol. (2016) 231:132–6. doi: 10.1016/j.vetpar.2016.04.011, PMID: 27117947

[25] Galliera E Tacchini L Corsi Romanelli MM. Matrix metalloproteinases as biomarkers of disease: updates and new insights. Clin Chem Lab Med. (2015) 53:349–55. doi: 10.1515/cclm-2014-0520, PMID: 25153404

[26] Li Y Liu H Xu L. Expression of MMP-9 in different degrees of chronic hepatitis B and its correlation with inflammation. Exp Ther Med. (2018b) 16:4136–40. doi: 10.3892/etm.2018.6673, PMID: 30344689 PMC6176163

[27] Cheng X Gao W Dang Y Liu X Li Y Peng X. Both ERK/MAPK and TGF-beta/Smad signaling pathways play a role in the kidney fibrosis of diabetic mice accelerated by blood glucose fluctuation. J Diabetes Res. (2013) 2013:463740. doi: 10.1155/2013/463740, PMID: 23936866 PMC3725803

[28] Cheng Z Limbu MH Wang Z Liu J Liu L Zhang X. MMP-2 and 9 in chronic kidney disease. Int J Mol Sci. (2017) 18. doi: 10.3390/ijms18040776, PMID: 28397744 PMC5412360

[29] Haberbosch W Gardemann A. Gelatinase B C(−1562) T polymorphism in relation to ischaemic heart disease. Scand J Clin Lab Invest. (2005) 65:513–22. doi: 10.1080/00365510500206575 16179285

[30] Zhang B Ye S Herrmann SM Eriksson P de Maat M Evans A. Functional polymorphism in the regulatory region of gelatinase B gene in relation to severity of coronary atherosclerosis. Circulation. (1999b) 99:1788–94. doi: 10.1161/01.cir.99.14.1788, PMID: 10199873

[31] Sampilvanjil A Karasawa T Yamada N Komada T Higashi T Baatarjav C. Cigarette smoke extract induces ferroptosis in vascular smooth muscle cells. Am J Physiol Heart Circ Physiol. (2020) 318:H508–h518. doi: 10.1152/ajpheart.00559.2019, PMID: 31975626

[32] Zhou L Le Y Tian J Yang X Jin R Gai X. Cigarette smoke-induced RANKL expression enhances MMP-9 production by alveolar macrophages. Int J Chron Obstruct Pulmon Dis. (2019) 14:81–91. doi: 10.2147/copd.S190023, PMID: 30587964 PMC6304243

[33] Le Y Cao W Zhou L Fan X Liu Q Liu F. Infection of *Mycobacterium tuberculosis* promotes both M1/M2 polarization and MMP production in cigarette smoke-exposed macrophages. Front Immunol. (2020) 11:1902. doi: 10.3389/fimmu.2020.01902, PMID: 32973788 PMC7468417

[34] Castro MM Rizzi E Prado CM Rossi MA Tanus-Santos JE Gerlach RF. Imbalance between matrix metalloproteinases and tissue inhibitor of metalloproteinases in hypertensive vascular remodeling. Matrix Biol. (2010) 29:194–201. doi: 10.1016/j.matbio.2009.11.005, PMID: 19969080

[35] Hansson J Vasan RS Ärnlöv J Ingelsson E Lind L Larsson A. Biomarkers of extracellular matrix metabolism (MMP-9 and TIMP-1) and risk of stroke, myocardial infarction, and cause-specific mortality: cohort study. PLoS One. (2011) 6:e16185. doi: 10.1371/journal.pone.0016185, PMID: 21283828 PMC3023803

[36] Tan J Hua Q Xing X Wen J Liu R Yang Z. Impact of the metalloproteinase-9/tissue inhibitor of metalloproteinase-1 system on large arterial stiffness in patients with essential hypertension. Hypertens Res. (2007) 30:959–63. doi: 10.1291/hypres.30.959 18049028

[37] Yasmin CM ME Wallace S Dakham Z Pulsalkar P Maki-Petaja K. Matrix metalloproteinase-9 (MMP-9), MMP-2, and serum elastase activity are associated with systolic hypertension and arterial stiffness. Arterioscler Thromb Vasc Biol. (2005) 25:372–8. doi: 10.1161/01.Atv.0000151373.33830.41, PMID: 15556929

[38] Cappuccio FP Siani A Barba G Mellone MC Russo L Farinaro E. A prospective study of hypertension and the incidence of kidney stones in men. J Hypertens. (1999) 17:1017–22. doi: 10.1097/00004872-199917070-00019, PMID: 10419076

[39] Taylor EN Stampfer MJ Curhan GC. Diabetes mellitus and the risk of nephrolithiasis. Kidney Int. (2005) 68:1230–5. doi: 10.1111/j.1523-1755.2005.00516.x 16105055

[40] Chung SD Chen YK Lin HC. Increased risk of diabetes in patients with urinary calculi: a 5-year followup study. J Urol. (2011) 186:1888–93. doi: 10.1016/j.juro.2011.07.011, PMID: 21944094

[41] Uemura S Matsushita H Li W Glassford AJ Asagami T Lee KH. Diabetes mellitus enhances vascular matrix metalloproteinase activity: role of oxidative stress. Circ Res. (2001) 88:1291–8. doi: 10.1161/hh1201.092042 11420306

[42] Liu Y Chen Y Liao B Luo D Wang K Li H. Epidemiology of urolithiasis in Asia. Asian J Urol. (2018) 5:205–14. doi: 10.1016/j.ajur.2018.08.007, PMID: 30364478 PMC6197415

[43] Dion M Ankawi G Chew B Paterson R Sultan N Hoddinott P. CUA guideline on the evaluation and medical management of the kidney stone patient—2016 update. Can Urol Assoc J. (2016) 10:E347–e358. doi: 10.5489/cuaj.4218, PMID: 28096919 PMC5234401

[44] Aykan S Tuken M Gunes S Akin Y Ozturk M Seyhan S. ApaL1 urokinase and Taq1 vitamin D receptor gene polymorphisms in first-stone formers, recurrent stone formers, and controls in a Caucasian population. Urolithiasis. (2016) 44:109–15. doi: 10.1007/s00240-015-0813-1 26275878

[45] Rendina D De Filippo G Gianfrancesco F Muscariello R Schiano di Cola M Strazzullo P. Evidence for epistatic interaction between VDR and SLC13A2 genes in the pathogenesis of hypocitraturia in recurrent calcium oxalate stone formers. J Nephrol. (2017) 30:411–8. doi: 10.1007/s40620-016-0348-8 27639591

[46] Rendina D Mossetti G Viceconti R Sorrentino M Castaldo R Manno G. Association between vitamin D receptor gene polymorphisms and fasting idiopathic hypercalciuria in recurrent stone-forming patients. Urology. (2004) 64:833–8. doi: 10.1016/j.urology.2004.05.013, PMID: 15491743

[47] Bu Q Zhu Y Chen QY Li H Pan Y. A polymorphism in the 3′-untranslated region of the matrix metallopeptidase 9 gene is associated with susceptibility to idiopathic calcium nephrolithiasis in the Chinese population. J Int Med Res. (2020) 48:300060520980211. doi: 10.1177/0300060520980211, PMID: 33345667 PMC7756046

[48] Wu Y Zhang J Li C Hu H Qin B Wang T. The activation of ROS/NF-*κ*B/MMP-9 pathway promotes calcium-induced kidney crystal deposition. Oxidative Med Cell Longev. (2021) 2021:1–20. doi: 10.1155/2021/8836355 PMC8208877 34211634

